# Decolonization in sexual and reproductive health research methods: a scoping review

**DOI:** 10.1186/s12913-024-11817-z

**Published:** 2024-11-25

**Authors:** Maya Stevens-Uninsky, Aisha Barkhad, Tonya MacDonald, Alexander Perez, Lawrence Mbuagbaw

**Affiliations:** 1 Department of Global Health, McMaster University, Hamilton, ON Canada; 2 Department of Health Research Methods, Evidence and Impact, McMaster University, Hamilton, ON Canada; 3 Independent Researcher, Washington, DC USA; 4 Department of Anesthesia, McMaster University, Hamilton, ON Canada; 5 Department of Pediatrics, McMaster University, Hamilton, ON Canada; 6 Biostatistics Unit, Father Sean O’Sullivan Research Centre, St Joseph’s Healthcare, Hamilton, ON Canada; 7 Centre for the Development of Best Practices in Health (CDPH), Yaoundé Central Hospital, Yaoundé, Cameroon; 8 Division of Epidemiology and Biostatistics, Department of Global Health, Stellenbosch University, Stellenbosch, South Africa

**Keywords:** Sexual and reproductive health, Reproductive health, Sexual health, Decolonization, Decolonized research, Decolonized methodologies, Community-centered research, Scoping review, Colonialism

## Abstract

**Background:**

As researchers and practitioners in the field of global health continue to acknowledge the ongoing impact of colonialism in their work, the call for decolonized research has increased. This has particular relevance in the field of sexual and reproductive health. Despite this recognized need, there is no singularly agreed upon definition of what it means to conduct decolonized research using decolonized methodologies. The aim of this review is to explore the approaches and methodologies used in contemporary sexual and reproductive health research aligned with decolonized systems of thinking.

**Methods:**

This review was developed and conducted in accordance with the JBI and the Extension for Scoping Reviews (PRISMA-ScR) Checklist. In January 2023, Medline (Ovid), Embase, EMCare, Global Health Database, and Web of Science were systematically searched for relevant studies. Relevant grey literature was also scanned. The screening and data extraction were conducted by four independent reviewers using an iterative approach. The findings were analyzed to uncover shared characteristics between the studies.

**Results:**

A total of 1775 articles were retrieved through our search strategy, of which 35 were included as discussing sexual health topics, and representing the principles of decolonization. Few of the included articles explicitly self-identified as decolonized literature. Common themes between studies included that most of the data collection was conducted in high-income countries, largely in North America, and the most prevalent sexual health topics were HIV/AIDs, and STIs/STDs. Most studies were qualitative, used community-based methodologies, and included some form of community advisory board.

**Conclusions:**

This scoping review identifies shared characteristics of both successes and gaps in decolonized research that may inform the methodological processes of future researchers. It emphasizes the need for more decolonized research originating in low- and middle-income countries, as well as decolonization of quantitative research methodologies. The findings also emphasize the importance of community engagement throughout the research process. A shared definition of decolonization is necessary to codify this body of work. Future researchers should focus on clearly communicating their approach in the methodology so that it can be replicated and become part of a shared definition.

International Registered Report Identifier (IRRID): DERR1-10.2196/45771

**Supplementary Information:**

The online version contains supplementary material available at 10.1186/s12913-024-11817-z.

## Background

As researchers and practitioners in the field of global health continue to acknowledge the ongoing impact of colonialism in their work, the call for decolonized research has increased [1]. Colonial influence on research is evident at a variety of points in the process, ranging from the determination of funding priorities and academic imperialism to the pervasive use of Western research methodologies and concepts in research design and implementation. Western concepts such as quantitative research tend to focus on experiments and principles of objectivity to uncover a singular truth or fact, limiting the presence of alternative world views [2]. The paradigms of Western research such as individualism, or positivism, have shaped the way contemporary research is conducted, how data is collected, and how knowledge is received, often overshadowing culturally specific and situationally relevant perspectives [3].

The root of decolonization lies in the process of reclaiming the power structures and cultures that were eroded or eradicated by colonialism [4]. When applied in a research methodology it prioritizes community leadership [5] and uproots imbalanced systems of power in research that are based in colonized institutions and epistemologies [6]. Scholars such as Battiste [7] and Smith [6] underscore that in decolonized research, the priority is for indigenous communities and their goals to take the lead, using culturally appropriate research paradigms to reclaim research and knowledge [3]. Therefore, truly decolonized research methodologies and methods will entail a critical examination of dominant Western methodologies [6]. This does not imply opposition to Western methods, but instead values dominant culture and culturally relevant research methodologies equally to determine what is most applicable and effective.

This scoping review pulls on the discourses of these prominent scholars of decolonization to identify important elements of decolonized research methodologies that are broadly agreed upon within the literature. These elements fit within three principles that create the foundation of decolonization in research. Therefore, for the purposes of this review, decolonized research is defined as research which contains elements of the following three key principles: (1) Research practices, from design through implementation, that place communities at the center of the work [6]; (2) Acknowledgement and/or critique of existing power imbalances that influence the research or topic of interest, such as colonialism, patriarchal systems, etc. [8]; and (3) Development of strategies that challenge Western research foundations and assumptions, or assess the extent to which they may or may not be appropriate [6].

Decolonized research methodologies are particularly crucial when it comes to sexual and reproductive health (SRH) issues. The institutions of colonialism have systematically oppressed women and sexual minorities, curtailing autonomy in decision making, particularly in relation to family planning. Colonial practices weaponized sexual health and reproduction as tools for population control [9], through the hyper-sexualization of indigenous or racialized women [10], gynecological experimentation, eugenics, forced sterilization [11], and homophobia [12], amongst others. This lasting legacy is reflected in contemporary history and ongoing practices of medical experimentation [13], as well as community mistrust of SRH services and institutions. Consequently, research on sexual and reproductive health topics has emerged as a critical focal point for this review.

Although many authors and researchers have begun to discuss the use of decolonized approaches to research and practice, there remains no singular understanding of how decolonization can be executed as a methodology, nor does there exist a set of best practice recommendations [14]. A shared definition is necessary to formalize decolonized practices and applications as the norm within global health research, and to ensure that research strategies are based on a clear foundation of evidence [15].

An overview of the shared principles within existing decolonized research is the first step towards an agreed upon definition of decolonized research and methodology. The aim of this scoping review is to identify contemporary sexual and reproductive health research aligned with decolonized systems of thinking and review the shared characteristics between them. The review asks the question “what are the commonly shared research designs, methods, and study characteristics of decolonized research methodologies in sexual health studies?”. The study will also identify ways in which researchers can learn from and replicate these shared characteristics in culturally appropriate and contextual ways, and where there are gaps that remain to be filled.

## Methods

This review was developed and conducted in accordance with the JBI [16] and the Extension for Scoping Reviews (PRISMA-ScR) Checklist. The initial search of literature was performed in January of 2023, using Medline (Ovid), Embase, EMCare, Global Health Database, and Web of Science. Grey literature sources were also searched, including World Health Organization (WHO), United Nations Children’s Fund (UNICEF), and others. Search strategies were developed using key terminology in decolonized sexual and reproductive health research, research methodologies and methods. Detailed search strategies can be found in the published protocol [17].

To be included in the review, studies needed to be: published between January 2012 and December 2022; have full-texts available; meet at least one element within each of the three principles of decolonization; and be focused on sexual and reproductive health. Additionally studies were included only if the primary participant in the study was a member of the community of focus; and if the study involved direct participant data collection. These inclusion criteria meant only primary data was included, rather than secondary analyses, or data collected from a secondary source, such as participants who were not the population of focus. Studies were excluded if they were a secondary analysis, or were abstracts, protocols, posters, book reviews, dissertations, or blog posts. There was no exclusion based on language or geographic area, as decolonized research can be conducted in any region or dialect.

A data extraction tool was developed for this scoping review. The tool was jointly developed between all the reviewers using strategies such as iterative discourse throughout the screening process, review of archetype articles, and multiple rounds of piloting. To enhance accuracy, agreement, and incorporate all perspectives, after each pilot round the authors met to discuss new findings and determine if data extraction questions should be added or removed. The authors endeavored to be reflexive during this process, prioritizing examination of personal and group biases and dynamics as a part of the review process. Relevant knowledge partners for this study are other researchers who might use these findings in their future research. As such, a range of academics and practitioners were engaged in discussion on the topic, which also informed the data extraction tool.

Screening and extraction were conducted using DistillerSR: Literature Review Software (Evidence Partners). To reduce selection bias, two reviewers screened each study at the abstract and full text level. Conflicts were resolved by discussion. Co-authors were consulted if a consensus could not be reached. Once included, data was extracted at two levels. Data were extracted by two of the four independent reviewers at each level. In the first level, data was extracted around: bibliometric data (such as author name, title, and year of publication); principles and elements of decolonization; sexual and reproductive health subject; and methodology. At the second level, data was extracted on: study characteristics; methods; outcomes; partnership; and community engagement. Missing data was flagged ‘not available’.

Topics were analyzed iteratively, with common themes and reflections identified throughout the data extraction process, and discussion between the authors. This led to an agile approach to descriptive analysis. Quantitative descriptive analysis was conducted using R and Excel.

## Results

**Fig. 1:**
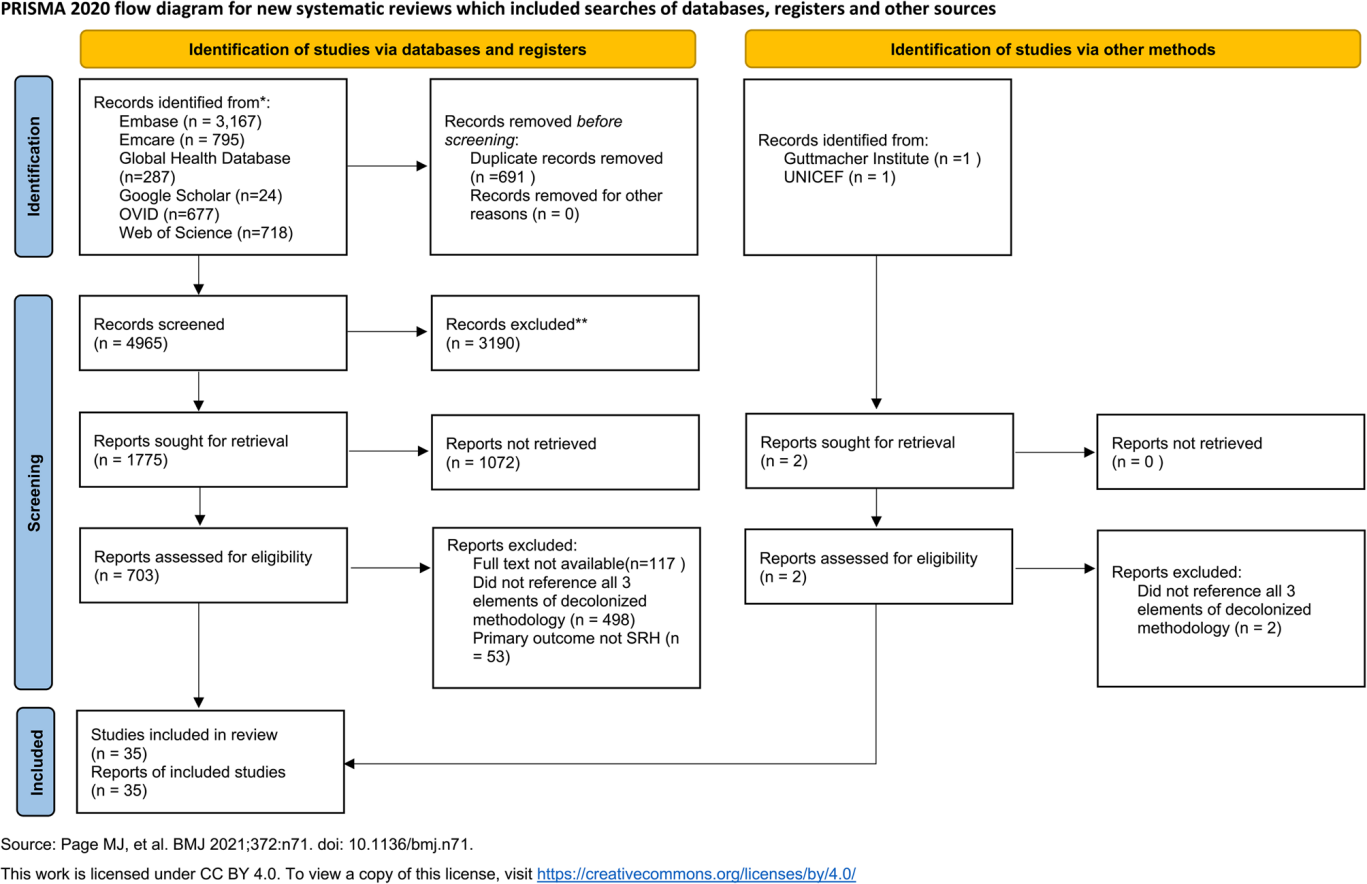
PRISMA diagram of the literature search

We identified 1775 studies through our initial search strategy, of which 703 were deemed potentially eligible after title and abstract review. Of these, 586 were available for full-text review. After completing our inclusion processes, we found that 35 met our inclusion criteria. The flow of studies is shown in the PRISMA diagram (see Fig. 1). A synthesis table of primary study characteristics can be found in Supplementary Table 1, Additional File 1.

### Study characteristics

#### Geographic location

**Fig. 2:**
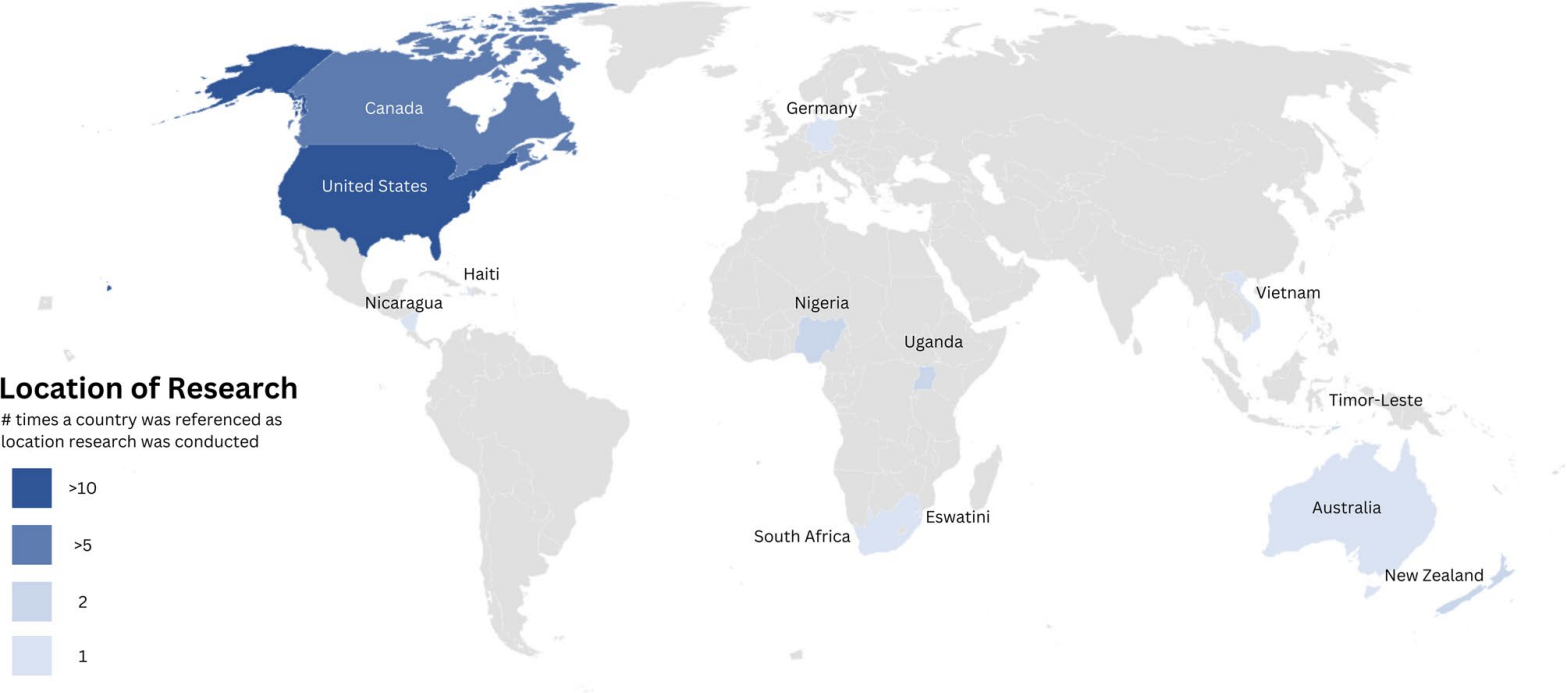
Location and frequency of research

Nearly two thirds (62.9%) of the studies were conducted in the Americas Region, as defined by the World Health Organization’s (WHO) region divisions. A further 14.3% were conducted in the African Region, and 11.4% in the Western Pacific Region. For more information on the most referenced countries, refer to Fig. 2.

68.6% of the studies were conducted in High Income Countries, according to the World Bank country income level classification [18]. Among the remaining studies, 8.6% were conducted in Low Income countries, 17.1% in Low-Middle Income Countries, and 2.9% in Upper-Middle. One study (2.9%) did research in countries with mixed income levels.

#### Sexual and reproductive health topic

**Fig. 3:**
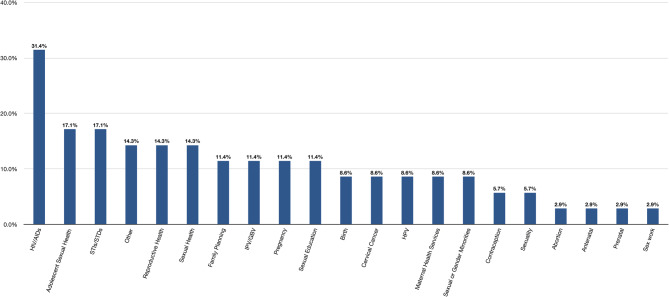
Sexual and reproductive health topic of interest in included studies

The SRH topic discussed most frequently across all the included studies was HIV/AIDs, which was identified as an outcome of interest in 31.4% of the included studies. 17.1% referenced adolescent sexual health and STIs/STDs as the SRH outcome of interest. The least commonly mentioned SRH topics were abortion, antenatal and prenatal care, and sex work, each of which were mentioned only once. The researchers identified several SRH topics that were not identified in any of the studies, including birth control, circumcision, female genital mutilation/clitoral cutting, and menstruation or menarche. Details can be found in Fig. 3.

#### Participant characteristics

The majority of the studies (65.7%) included young adults, age 18–39. Almost half (45.7%) included middle-aged participants (40–59), and 31% included older adults (60+). Only 3 studies (8.6%) included adolescents (13–17), and none included children.

All but one study identified the sex or gender of their participants. The overwhelming majority of the studies (82.9%) included women in their study population, while over half (51.4%) included men. Only 1 study referenced gender non-conforming or trans-identifying populations.

Fifteen studies (42.8%) identified their priority population as Indigenous. Six of the 15 (40%) identified Indigenous Research Methods in their study design. Of the 15 studies, 11 (73.3%) were conducted in the Americas region, and 4 (26.6%) were conducted in the Western Pacific. By contrast, none of the studies in Africa explicitly identified an Indigenous population. For further detail, see Table 1.

**Table 1:** Location of indigenous research studies

	Africa	America	Europe	Multiple	South East Asia	Western Pacific	Total
**Total # Studies**	5	22	1	1	2	4	35
**Identifying Indigenous Population % (n)**	0.0% (0)	50.0% (11)	0.0% (0)	0.0% (0)	50.0% (1)	75.0% (3)	42.8% (15)
**Studies using Indigenous Research Methods % (n)**	0.0% (0)	9.1% (2)	0.0% (0)	0.0% (0)	150.0% (1)	75.0% (3)	40.0% (6)

### Decolonization

**Fig. 4:**
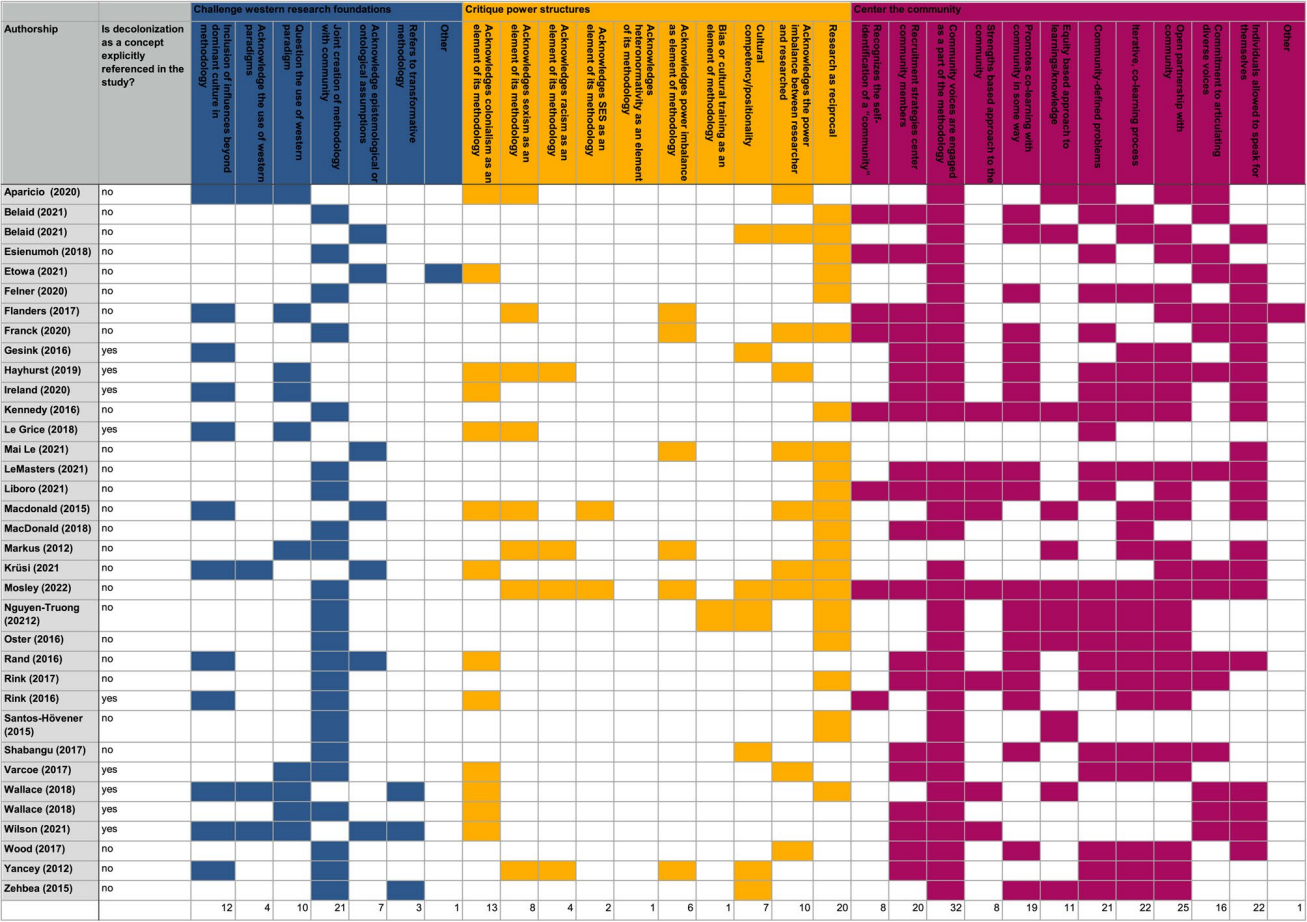
Distribution of common elements of decolonization

To be included in this review, studies had to exemplify all three identified principles of decolonized research: (1) strategies challenging Western research foundations; (2) critiques of power structures; and (3) a community-centered approach. Each of these principles was divided into sub-elements which allowed reviewers to detail how the study exemplified each principle. Figure 4 shows the prominence of each principle and identifies their constituent elements. See Supplementary Tables 7–9, Additional File 1 for definitions of each element.

Regarding the principle of “Challenging Western research foundations”, the most prevalent element was ‘Joint creation of a methodology with the community’, present in 60% of studies. None of the studies identified all 7 elements of this principle. Regarding the principle of “Critiquing power structures”, the most commonly identified element was ‘research as reciprocal’, present in 57.1% of studies. None of the studies used all 12 elements of this principle. Finally, in the principle “Centering the community”, 91.4% of studies identified ‘community voices engaged as part of the methodology’. One study in this category used all 12 elements of this principle of decolonized research.

Only 9 studies (25.7%) explicitly used the term “decolonization” in their publication, 2 of which were separate publications by the same author on the same research. All 9 of these studies were published after 2017, and the majority (7 out of 9) mentioned decolonization in their methods section. Only one-third mentioned decolonization in more than one section. Table 2 offers a review of the context in which “decolonization” is employed in these articles.

Few of these 9 studies defined the meaning of decolonization or its application in their work. Those who did define their use of “decolonized” focused on centering the needs and perspectives of the community or Indigenous peoples with whom they worked [19–21]. Other mentions of decolonization focused on the process of decolonizing the research, such as through community partnership [22], or the relationship to existing but external decolonization efforts or methodologies [23–25].

**Table 2:** References to decolonization in included studies

Authorship	Mention of Decolonization	Key Phrase
Gesink et al., 2016	Methods	“Cree and non-Cree partners co-led the research as part of the **decolonizing** and indigenizing research”
Hayhurst et al., 2019	Conclusion	“…prevention of violence to the land and physical environment is deeply enmeshed with preventing violence against young women’s bodies, and to broader **decolonization** efforts”
Ireland, S., Maypilama, E. L, 2020	Abstract, Methods, Results, Discussion, Conclusion	“We used a **decolonising** participatory action research (PAR) methodology. Our approach explicitly prioritised Yolŋu ways of being, doing and seeing.” “This is a **decolonising** learning approach asserting Yolŋu sovereignty over their knowledge systems, physical bodies and ancestral lands.”
Le Grice, J., Braun, V., 2018	Abstract, Methods, Results, Discussion, Conclusion	“Mana Wāhine research privileges Māori women’s analyses and aspirations, seeking to **decolonise** historical and contemporary colonial interpretations about Māori” “**Decolonising** these assumptions, by speaking to the influence of colonisation on Māori cultural ways of being and practices”
Rink et al., 2016	Methods	“CBPR may be viewed as a **decolonizing** research methodology that is responsive to promoting cultural relevancy and empowerment.”
Varcoe et al., 2017	Methods, Analysis	“Our process to adapt iHEAL for Indigenous women in urban contexts was underpinned by critical theoretical and **decolonizing** approaches.”
Wallace et al., 2018	Methods	“We employed a qualitative research design with **decolonizing** methodology. This means we placed Indigenous knowledge in the center of our research and considered respectfully Timor-Leste’s history of colonization throughout our projects.” ” **Decolonizing** methodologies must be beneficial and empowering for the Indigenous participants” “We are mindful of the tension that exists between our **decolonizing** methodology and inductive coding when compared with a Western biomedical framework”
Wallace et al., 2018	Methods	“The researchers used a **decolonising** methodology, situating Timorese voices and worldviews in the centre of the research process, and Timorese guidance, collaboration and interpretation occurred across all phases of the project “
Wilson et al., 2021	Methods	Kaupapa Māori research methodology draws on a Māori worldview and **decolonization** and intersectionality theories to inform the analysis and interpretation of the data”

Of these 9 studies, 5 used an Indigenous Research Design, 3 were qualitative, and the remaining study employed a mixed methods design. Of the 35 studies meeting our inclusion criteria, only 6 used Indigenous Research Design, indicating there may be a relationship between prioritizing Indigenous Research Methods where appropriate, and taking a decolonial approach to research.

Data were also collected on the number of studies which included a positionality statement to reflect on researcher standpoint or potential biases in relation to the research. Of the 35 included studies, 5 included a positionality statement, 2 of which explicitly mention decolonization.

### Study design and methodology

#### Methodology

Most of the included studies (63%) used a qualitative design, with another 17% using Indigenous Research Methods, 11% mixed methods designs, and 9% quantitative.

Across all studies, the two most frequently used research methodologies were Community Based Participatory Research (CBPR), and Participatory Action Research (PAR), each used in 37% of the included studies. Both are broadly identified as qualitative research methodologies, although one quantitative study identified CBPR as a part of its methodology. Other frequently used methodologies were Decolonized Methodology and Post-Colonial Research, both identified in 11% of the studies.

Among qualitative studies, CBPR and PAR were the most commonly used research methodologies, present in 50% and 40.9% of studies respectively. However, the majority (83.3%) of Indigenous Research Design studies used Indigenous Research Methodologies specific to the community in which they were working, including Cree research protocol and ethics, Two-Eyed Seeing, Piliriqatigiinniq Partnership Community Health Research Model, Mana Wāhine (Māori feminist), Kaupapa Maori, and Inuit Qaujimajatuqangit. Half of the Indigenous Research studies also used Decolonized Research Methods.

Three of the 4 mixed methods studies identified PAR research methodologies, and 25% identified CBPR research methodologies. These studies also included quantitative research methodologies, with cross-sectional methodology, experimental methodology, and quasi-experimental methodologies each getting one mention. In the quantitative studies, each of the following research methodologies was referenced once: cross-sectional study methodology [26], ecological study methodology [27], and experimental methodology [28]. Details of designs and methodologies of included studies can be found in Supplementary Tables 2–6, Additional File 1.

#### Data collection methods

**Fig. 5:**
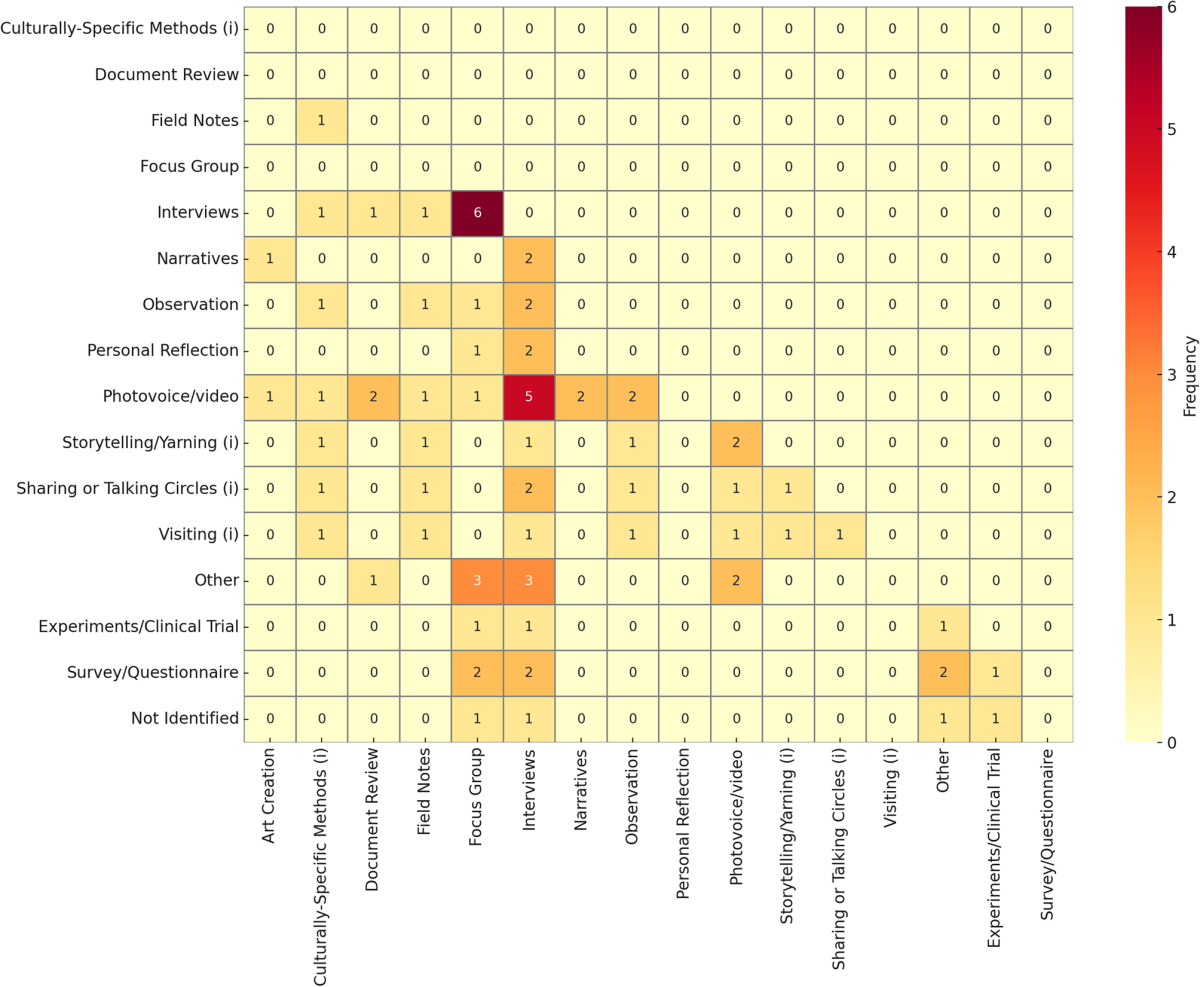
Data collection methods most commonly used together

Most studies involved multiple forms of data collection methods. The most common form was interviews, used in 60% of studies, focus groups, mentioned in 31% of studies, and photovoice, which was referenced in 29% of studies. 17% of studies employed the use of a survey or questionnaire. All the studies which used a survey were mixed methods or quantitative. The average number of data collection methods used per study was 2.1, with 74% of studies only using 1 or 2 methods to collect data. The most common combination of methods was using focus groups alongside interviews, followed by a combination of interviews and photovoice (Fig. 5).

None of the included studies referenced the development or adaptation of a new data collection tool or method for the purposes of the study, although many of the methods were chosen with input from members of the community to reflect local knowledge translation procedures and cultural norms. This is in part represented by the 20% of studies which explicitly identified and used Indigenous Research Methods such as talking circles or yarning.

None of the studies mentioned a process of making changes to the standard data collection methods to make it more relevant to the research and context. For example, survey questions may have been developed and modified for the research, but the process of delivering a survey remained standard to the norm. For more detail on of study designs, methodologies, and data collection methods used, see Supplementary Tables 2–6, Additional File 1.

#### Data analysis

Within the 94.2% of studies that detailed their data analysis process, 78.8% mentioned the researchers participating in the analysis. In 33% of the studies, data analysis was conducted exclusively by the researchers with no other participants. The other group most frequently involved in data analysis were community members, who assisted with the analysis in 21.2% of studies. 9.1% of studies included local research assistants in the data analysis process. In only 1 study were researchers not involved in the analysis at all.

### Community participation in research

#### Partnerships

One measure of community participation in research was through partnership with community members or organizations. 22 of the 35 studies (66.3%) mentioned partnerships. All but one of these partnerships involved an academic institution. The most frequent partnership was between academic institutions and community organizations. The only study that did not involve an academic institution was a partnership between an NGO and an international organization [23].

Sixteen of these 22 studies (72.7%) had a bi-lateral partnership, usually between an academic institution and community organization (31.3%), or academic institution and community members (31.3%). Four of the studies had a three-way partnership, all of which were between academic institutions, community organizations, and community members.

#### Community engagement points

There are several strategies for community engagement and involvement in the research process. We identified 16 points throughout the research process where community engagement commonly occurs. See Table 3 for a chronological arrangement of these points in the research process. Of the 35 studies, 4 (11.4%) engaged with the community at 9 or more points in the research process, with another 13 (37.1%) engaging community members at 2 or fewer points in the research.

Community members were engaged at the data collection point of the research process in 54.3% of the studies. Community members were least likely to be involved in the background research (2.9%). 25.7% of studies included community members at all stages (beginning, middle, end) of the research process. Community members were least likely to be involved at the beginning of the research. Table 3 contains the details regarding points of community involvement.

**Table 3:** Points of community engagement in the research process

Stage of Research Process	Points of Community Involvement	Number of Studies (*n*)	Points of engagement identified in Studies (%)	
**Beginning**	**Proposal writing**	5	14.3%	60.0%
	**Background research**	1	2.9%	
	**Identifying the problem/need for intervention or research**	7	20.0%	
	**Research question**	6	17.1%	
	**Research design**	13	37.1%	
	**Research methods development**	7	20.0%	
	**Selection or design of data collection tools**	14	40.0%	
	**Definitions of successful outcomes**	3	8.6%	
**Middle**	**Implementation**	6	17.1%	71.4%
	**Recruitment**	8	22.9%	
	**Collecting data**	19	54.3%	
**End**	**Data analysis**	17	48.6%	65.7%
	**Member checking**	13	37.1%	
	**Knowledge translation**	13	37.1%	
	**Not mentioned**	8	22.9%	
	**Other**	1	2.9%	

#### Community advisory roles

Another characteristic that was used to identify community engagement and participation was the presence of a community advisory board or committee (CAB). The role of a CAB is to incorporate the voice of community members in a more involved and informed way throughout research activities, and can be an indicator of in-depth community engagement [29]. Of the 35 included studies, 16 (45.7%) referenced convening some form of CAB.

**Fig. 6:**
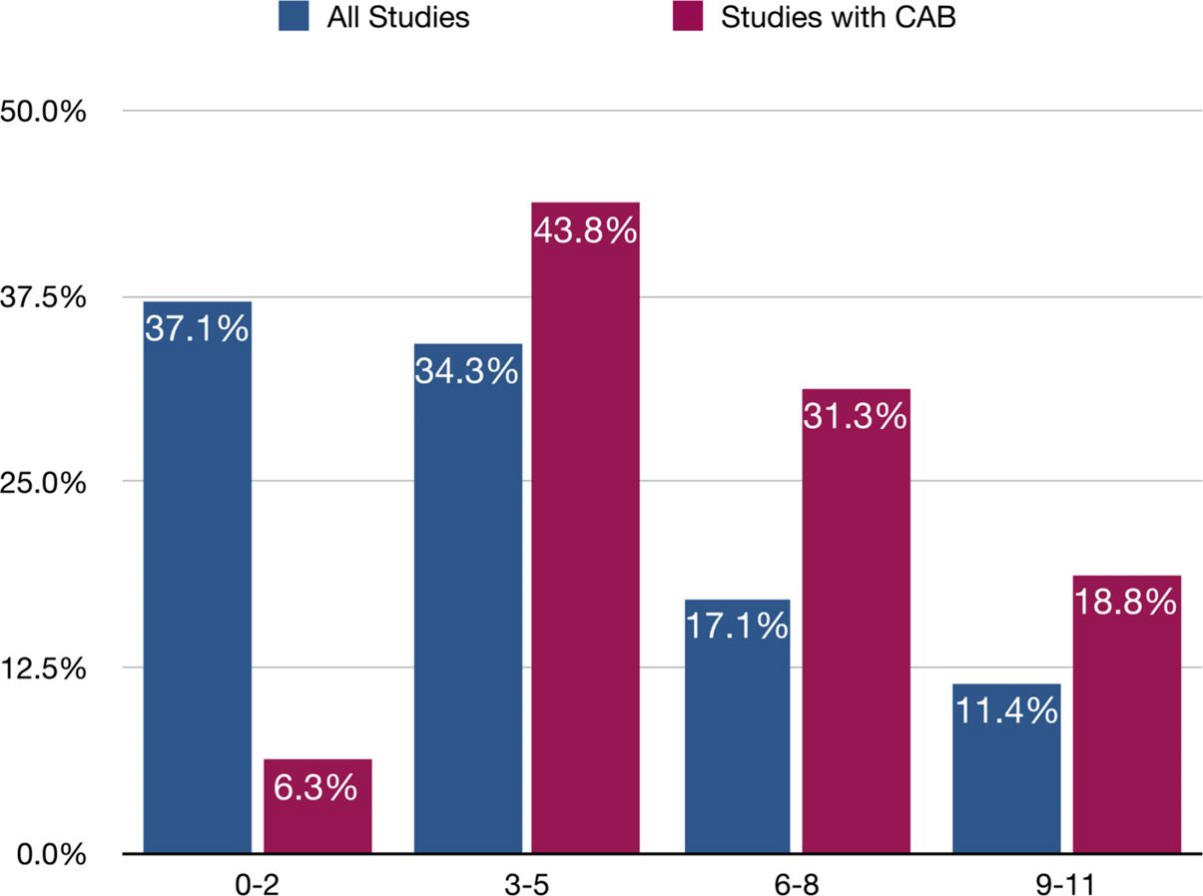
Number of points of engagement in all studies vs. studies with community advisory boards

It is also of note that in studies with CABs, community members were engaged in the research process substantially more than in studies without CABs. See Fig. 6 for details. The same is true regarding the extent of community involvement. Studies with a CAB were on average 20% more likely to include community members at all points of the study (beginning, middle, and end), but particularly more likely to include them earlier on in the study.

## Discussion

This review explores the use of decolonized research principles in SRH research, to identify areas for learning as well as areas for growth. We identify some commonly shared characteristics in the way research is conducted, as well as numerous challenges in identifying a pedagogy for conducting decolonized research. The study describes elements of the included research ranging from bibliometric details to methodologies and methods. We identified three key themes in the way decolonized SRH research is being conducted, and ways in which it can continue to evolve. These include: (a) the need for a shared definition and approach to decolonized research; (b) the need for decolonized research to explicitly redress power imbalances; and (c) the need for researchers to incorporate the principles of decolonization at a foundational level.

### Finding a shared definition and approach to decolonization

Our study exemplifies the lack of a shared definition or approach to decolonization. Few studies included in this review used the word decolonization in any form, and even fewer defined or detailed how the terminology was applied to their research or methodology. This may be due to the current lack of guidance and definition of the term creating reluctance to identify research as meeting a set of unknown criteria implied by “decolonized methodology” [14].

Some shared characteristics in existing research provide direction to other researchers in designing and utilizing a shared decolonized methodology. The majority of studies used methodologies which take a community-based approach to research (such as CBPR or PAR) that is rooted in intersectionality [30], prioritizing and amplifying community voices, and challenging existing power imbalances [31].While the application of these methodologies was not uniform across studies, it is nonetheless an indicator that collaborative community approaches are a central element of decolonized methodologies.

There is also a noteworthy lack of decolonized research practices when conducting quantitative research. The relative absence of decolonized quantitative research suggests a direction for further research. It is worth considering whether quantitative research methodologies encourage an assumption that their scientific nature removes the need to account for systematic imbalances or implement culturally appropriate methods. Also possible is the assumption that it may be unduly challenging, if not unnecessary, to decolonize the more formal tools and strategies of quantitative research. However, quantitative data is not immune to the pitfalls of colonial history and power imbalances, and also requires decolonization [32]. In fact, given the historical relationship between Western scientific paradigms, the experimentation done on colonized peoples, and the ongoing health research conducted on colonized and marginalized groups with little input from participants, this is a vital area for future work [33].

A clear shared definition of decolonized research and its methodologies would facilitate its application in both quantitative research, where community centered methodologies are less frequently applied, and qualitative research where community research is applied without being identified as an element of decolonization. By clarifying personal and academic definitions and applied strategies of decolonized research in publications, we will come closer to a shared pedagogy and greater ease of application of both the term and its methodologies.

### Redressing power imbalances

A critical element of decolonization, particularly in research methodologies and design, is the acknowledgment, critique, and counterbalancing of unequal or dominant systems of power [6, 7]. The current process of global health research contains numerous systemic power imbalances rooted in a history of colonial influence. Power imbalances emerge throughout the entirety of the research process from funding availability, prioritization of research topics, objectives, and questions, to data analysis, and the dissemination of study findings [34]. The location and topic of research is strongly influenced by the epistemologies and values of dominant culture researchers and funding institutions [35].

The authors identified the explicit mentions of colonialism and its lasting impact on the community or research in question as a method for analyzing power imbalances in the included studies. The nuance lies not merely in the mention of colonial elements as a matter of background, but in identifying how it is accounted for in the research methodology, including the manners in which multi-faceted inequalities are addressed [36]. This approach to redressing power imbalances was only present in 37% of the included studies and denotes a gap in decolonized literature. This lack of acknowledgement of colonialism’s impact affects the quality of data and eventual SRH outcomes, leading to insufficient consideration of intersecting inequities in race, sex, gender, and other areas, all of which negatively impact health outcomes and cannot be overlooked [37]. Identifying, acknowledging, and accounting for these disparities is a vital part of decolonized SRH research moving forward.

Geographic distribution or income level of the area where research is conducted is another space where the field of decolonized research must work to redress power imbalances. The limited number of studies conducted with decolonized principles in LMICs represents a substantial shortfall. The application of these research principles should be at parity between HICs and LMICs, as both continue to carry the structural power imbalances of colonial history. This is particularly relevant in relation to SRH research, where there is clear evidence of a colonial history of systemic oppression through medical and sexual interventions across all formerly colonized nations regardless of income level [13].

Common themes in redressing power imbalances were also identified in the SRH topic areas. The majority of included studies involved SRH topics such as HIV and other STI/STDs [38]. HIV/AIDs in particular is a SRH topic that often reflects a history of stigma and oppression against communities who have limited systemic power to address their needs [39], which aligns with the principles of decolonization. However, there was a gap in research conducted on topics specific to vulnerable population groups. Trans or non-conforming participants were under-represented, as were SRH topics such as sex-work. As a decolonized approach is intended to support those who have been marginalized, providing them with a voice, this is a notable oversight and should recommend opportunities for future research. Research that prioritizes building trust and bridging the gaps between community and global health institutions is of particular importance.

The involvement of the community at early stages and throughout the research process is one identified strategy for redressing the inherent power imbalance between the researcher and the “researched” [40]. Among the included studies, research participants were rarely included at the beginning stages of the research, where directional decisions were being made. This lack of early engagement should be addressed in future research. The current process of research design and implementation, including grant writing and institutional ethics approval, creates barriers for early community engagement and speaks to the need to reconsider power imbalances in current research systems. To truly even the playing field between community members and researchers, all should be involved at all points in the process.

A final common characteristic between studies was the frequency of community partnerships, and CABs. These community engagement strategies provide future researchers with knowledge that will guide them towards greater equity in decision-making power between the community and researchers. One recommendation for future researchers is to make explicit the terms, responsibilities, and outcomes of community partnerships or advisory boards thereby providing resources and guidance to other researchers hoping to accomplish similar work.

These forms of shared decision-making are critical to redressing power imbalances and will help lead to more equity in decision-making power between the community and the researchers. The next step for decolonized research is to explore ways in which researchers can begin supporting community members and organizations as leaders in the research. As it stands, the final decision-making in research almost always lies with the researcher, and this represents an inherent and long-standing power imbalance. These shared characteristics point towards the need for, and development of, a strategy to redress the inequal power hierarchies present in traditional research rooted in a Western paradigm.

### Addressing superficial adherence to decolonizing principles

A final theme shared by the included studies was reference to decolonized methodology or community engagement without clarifying how these approaches were incorporated throughout the research. This shared characteristic of “lip service” to decolonization was identified in multiple areas of the included studies.

One way in which this was represented was in the terminology used by authors. In CBPR and PAR studies, the commonly accepted standard practice in the methodology is democratic participation of the community at all stages in the research process [41, 42]. Even though CBPR and PAR were used in the majority of the studies in this review, specific details of community participation were lacking. It is therefore plausible that the language of these methodologies was used nominally, but the approach was not executed to the fullest possible extent.

A further shared characteristic can be found in the data collection methods of the included studies. None of the included studies developed or adapted new data collection tools. The most commonly referenced data collection tools were often rooted in dominant culture methodology and may have been inappropriate for the research setting. To gather valid data appropriately, it is essential to decolonize the methods themselves [31]. Culturally appropriate, minimally intrusive, and narrowly tailored data collection instruments should be prioritized by researchers.

Subsequent research must make efforts to detail in the text the ways in which the data collection methods being used are tailored or are already appropriate for the context. This will provide guidance for other researchers. Researchers should also consider developing and advocating for the development of culturally appropriate tools or adaptations specific to the location or research they are undertaking, to diversify the data collection methods available and in common use.

Most of the studies did not describe their implementation of the decolonized elements they referenced. For many of the studies, it was the terminology around decolonized methodology that was discussed, rather than the execution. Sharing experiences and explanations about implementing decolonized research is vital in encouraging its presence and guiding others. To prioritize decolonized research methodologies in a clear and transparent matter is a vital step toward encouraging its widespread replication.

For future research, it is clear that “lip service” to decolonized research methodologies is not adequate in decolonizing outcomes. Researchers must find more ways to not only emphasize the community’s role and involve them early and often, but also to encourage and support other authors by detailing the implementation of decolonized methodologies in their work, so others can learn, and research norms can shift.

## Strengths and limitations

One of the strengths of this study is that it was conducted by a multi-lingual, international, and interdisciplinary team of global health practitioners and researchers, with a broad range of experiences across a variety of relevant sexual health topics. A data extraction tool was also developed specifically for this review, which created a nuanced and specific set of data held to stringent criteria. In addition, a rigorous procedural and methodological approach was applied to this scoping review. The reviewers met frequently, and regularly discussed and recorded findings and kept notes as the research proceeded, taking an iterative approach to the data extraction throughout the various levels. This approach meant that data collection process was refined and better calibrated over time, mitigating, among other impacts, potential subjectivity of the content analysis.

One limitation of these findings is that this review may under-represent the true body of decolonized work available, as studies were identified using traditionally Western terms, within a Western institutionalized context. This is an issue inherent in doing decolonized work within a dominant culture institution. Another limitation is the fact that while publications in any language could be included, search terms were only in English, which may have limited the scope of the findings. Multiple studies in other languages were identified through the search, but were excluded exclusively based on the inclusion criteria, so it is the author’s opinion that this was not a significant limitation. A final limitation was the lack of pre-existing definition for decolonized research and methodologies. This was addressed with the development of a multi-faceted definition based on the work of recognized researchers in the field of study.

## Conclusion & future directions

The importance of decolonized research is increasingly recognized in the academic space. However, without a shared definition, direction, and approach, it is challenging for researchers to apply and discuss as a concept. Our review demonstrates that there is a compelling need to identify a shared definition and pedagogy for decolonization; use this research approach to redress power imbalances in global health research; and take research terminology from concept to practice. It is encouraging that many researchers examining SRH through a decolonized lens are challenging Western research foundations, critiquing existing power structures, and centering and engaging the communities they work in through their research. Nonetheless, there are several recommendations in research and practice that can be taken to future work.

A common methodology and approach to decolonization of research should be clearly defined and widely disseminated. While this scoping review attempts to contribute to a shared pedagogy, it has also identified the need to create a common understanding of decolonization that can be applied in qualitative and quantitative literature. One approach is to learn further from the Indigenous Research Methods and Methodologies frequently represented in the included studies. While there are many versions of Indigenous research, common elements between them are a shared history of colonial intervention, self-determination and agency, and the inclusion of non-Western epistemologies and ontologies [31]. Utilizing non-dominant culture research design and methodologies contributes towards the need to consciously redress power imbalances in Western research processes.

Another key recommendation emerging from this study is the need for greater transparency and knowledge sharing when it comes to decolonized approaches to research. The inclusion of strategies and methodologies of decolonized research within published studies serves to not only emphasize its value and relevance, but also standardizes the practice and facilitates it for future researchers. Researchers should focus on elaborating upon their methodologies for decolonized research to assist others in the field.

Finally, this study recommends that researchers working towards a decolonized research methodology should focus on cultural relevancy and appropriateness in order to support the dismantling of residual colonial power structures. Methods such as community partnership, more nuanced and diverse standards and tools for data collection, cultural navigators for research, and other strategies will help to create research that best serves the needs of communities over all else.

The findings of this scoping review show the importance of continuing to discuss, utilize, and share information around the meaning and applicability of decolonized research methodologies and methods. By summarizing these findings on the shared characteristics of decolonized SRH research the authors are able to draw initial implications for future research and inform the design of proposed studies.

## Electronic supplementary material

Below is the link to the electronic supplementary material.

Supplementary Material 1.

## Data Availability

The datasets used and/or analysed during the current study are available from the corresponding author on reasonable request.

## Abbreviations

CAB: Community Advisory Board
CBPR: Community-Based Participatory Research
HIV/AIDS: Human Immunodeficiency Virus/ Acquired Immunodeficiency Syndrome
JBI: Joanna Briggs Institute
NGO: Non-Governmental Organization
PAR: Participatory Action Research
PRISMA: Preferred Reporting Items for Systematic Reviews and Meta-Analyses
SRH: Sexual and Reproductive Health
STI/STD: Sexually Transmitted Infection/Disease
UNICEF: United Nations International Children’s Emergency Fund
WHO: World Health Organization
