# Supplementary material for: Decolonization in sexual and reproductive health research methods: a scoping review
Source: BMC Health Serv Res. 2024 Nov 25;24:1460. doi: 10.1186/s12913-024-11817-z (PMC11587638; doi:10.1186/s12913-024-11817-z)
Supplement: Supplementary file 1 — Supplementary Material 1. [file 12913_2024_11817_MOESM1_ESM.docx]

Table S1: Synthesis table of primary study characteristics of all included articles

| **Authors** | **Year of Publication** | **Study Location** | **Author Location** | **Study Design** | **Methodology** | **Methods** | **SRH Outcome** | **Priority population** | **Primary Objective** |
| --- | --- | --- | --- | --- | --- | --- | --- | --- | --- |
| Aparicio, E. M., Kachingwe, O. N., Phillips, D. R., Jasczynski, M., Cabral, M. K., Aden, F., Parekh, E., Espero, J., Childers, C. | 2020 | US | USA | Qualitative | Participatory action research | Photovoice/video | Adolescent Sexual Health, Family Planning, Reproductive Health, Sexual/Reproductive Education, Sexual Health | youth experiencing homelessness | Discuss experiences of young homeless women in a program, and the impact on waiting to start or expand their families, and homelessness. |
| Belaid, L., Atim, P., Atim, E., Ochola, E., Ogwang, M., Bayo, P., Oola, J., Wonyima Okello, I., Sarmiento, I., Rojas Roza, L., Zinszer, K., Zarowsky, C., Andersson, N. | 2021 | Uganda | Canada | Mixed Methods | Participatory action research | Focus Group, Interviews, Fuzzy Cognitive Mapping, Deliberative Dialogue, Survey/Questionnaire | Pregnancy, Perinatal Health | pregnant women | Describe participatory codesign of interventions to improve access to perinatal care services in Northern Uganda. |
| Belaid, L., Atim, P., Ochola, E., Omara, B., Atim. E, Ogwang, M., Bayo, P., Oola, J., Okello, I. W., Sarmiento, I., Rojas Rozo, L., Zinszer, K., Zarowsky, C., Andersson, N. | 2021 | Uganda | Canada | Mixed Methods | Grounded theory, Participatory action research, Cross-sectional study | Focus Group, Fuzzy Cognitive Mapping, Deliberative Dialogue, Survey/Questionnaire | Birth, Family Planning | women and families needing perinatal care | to explore women and community views about the causes of short birth intervals to inform a culturally safe approach to child spacing in Nwoya district in northern Uganda. |
| Esienumoh, E.  E., Allotey, J., Waterman, H. | 2018 | Nigeria | Nigeria | Qualitative | Participatory action research | Focus Group, Interviews, Observation, Photovoice/video | Maternal Health Services, Pregnancy, Maternal Mortality | women of childbearing age | To facilitate the empowerment of members of a rural community to plan to take action to prevent maternal mortality |
| Etowa, J., Ekanem, E., Ariyo, O., Inoua, H., Ashiri, Y., Nare,H., Akan Essien, E., Etowa, E. B. | 2021 | Canada, Nigeria, and USA | Canada | Qualitative | Community based participatory research, Ethnography, Post-Colonial research | Interviews | HIV/AIDs | African, Caribbean, and Black mothers living with HIV | examine and understand the ethnocultural context of infant feeding practices and experiences of childbearing HIV positive Black women during the first year of infant life (infancy period). |
| Felner, J.  K., Kieu, T., Stieber, A., Call, H., Kirkland, D., Farr, A., Calzo, J. P. | 2020 | USA | USA | Qualitative | Community based participatory research | Document Review, Interviews, Photovoice/video | STIs/STDs (Hepatitis A) | transitional aged youth (TAY, aged 18–24) experiencing/ed homelessness | Examine the social and environmental determinants of the HAV outbreak and health among TAY. |
| Flanders, C. E., Ross, L. E., Dobinson, C., Logie, C. H. | 2017 | Canada | USA | Qualitative | Community based participatory research | Focus Group | Sexual or Gender Minorities, Sexual Health | Young bisexual and other nonmonosexual people who self-identify as women, cisgender and transgender inclusive | to develop a better conceptual understanding of what factors young bisexual women see as related to their sexual health |
| Franck, L. S., McLemore, M. R., Williams, S., Millar, K., Gordon, A. Y., Williams, S., Woods, N., Edwards, L., Pacheco, T., Padilla, A., Nelson, F., Rand, L. | 2020 | US | USA | Qualitative | Research Priorities of Affected Communities protocol | Focus Group | Birth, Maternal Health | women of color living in three communities that experience disproportionately high rates of preterm birth | Include Women of color  in setting the agenda regarding preterm birth research. |
| Gesink, D., Whiskeyjack, L., Suntjens, T., Mihic, A., McGilvery, P. | 2016 | Canada | Canada | Indigenous Research Methods | Community based participatory research, Community-led research, Cree research protocol and ethics | Interviews | IPV/GBV, Sexuality, STIs/STDs | members of Saddle Lake Cree Nation, an Indigenous community in Alberta, Canada | to understand the context, issues, and beliefs around high STI rates from a nêhiyaw (Cree) perspective |
| Hayhurst, L. M. C., Centeno, L. D. C. | 2019 | Nicaragua | Canada | Qualitative | Feminist theory, Participatory action research, Post-Colonial research | Interviews, Photovoice/video | IPV/GBV | young women from Nicaraguan community | to unpack the connections between gender-based violence and the environment in sport, gender and development (SGD) programming in Nicaragua |
| Ireland, S., Maypilama, E. L. | 2020 | Australia, Indigenous | Australia | Indigenous Research Methods | Decolonized Methodology, Participatory action research | Culturally-Specific Methods (i), Field Notes, Interviews, Observation, Photovoice/video, Storytelling/Yarning (i), Sharing or Talking Circles (i), Visiting (i) | Adolescent Sexual Health, Reproductive Health, Sexual/Reproductive Education | Female Yolŋu | This paper demonstrates one approach to developing a reproductive health literacy framework for Yolŋu (Indigenous) women in a remote Northern Australian setting. |
| Kennedy, T., Teti, M., Hayes, D., Pichon, L. C., Farnan, R. | 2016 | USA | USA | Qualitative | Community based participatory research,  Ethnography | Interviews, Narratives, Photovoice/video | HIV/AIDS | women with HIV  (USA) | to explore the effect of selfportrait taking on women who chose to show their face in public photovoice portraits by identifying why women took self-portraits and how the self-portrait taking affected them. |
| Le Grice, J., Braun, V. | 2018 | New Zealand | New Zealand | Indigenous Research Methods | Decolonized Methodology, Feminist theory, Mana Wāhine (Māori feminist) | Interviews | Sexual/Reproductive Education, Sexuality | Maori women and men from New Zealand | explore examples of Māori sexual health psychologies informed by mātauranga Māori and ask what areas of mātauranga Māori can we legitimate and what areas of colonising assumptions can we deconstruct, in the provision of good quality sexuality education. |
| Le, T. M., Yu, N. | 2021 | Vietnam | Vietnam | Qualitative | Participatory action research | Document Review, Photovoice/video | Adolescent Sexual Health, Reproductive Health, Sexual Health | Vietnamese ethnic minority unmarried girls (in Vietnam) | to address the question: what are the sexual and reproductive health challenges facing young minority ethnic girls living in the Northern Uplands of Vietnam? |
| Lemasters, K., Atkins, K., Oloonabadi, S. A., Munn, T., Eng, E., Lightfoot, A. F. | 2021 | US | USA | Qualitative | Community based participatory research | Focus Group, Photovoice/video | HIV/AIDs, Sexual or Gender Minorities | Black men who have sex with men (MSM) and living w HIV | understanding the daily lives of Black MSM on PrEP in the Triangle Region of North Carolina, including barriers and facilitators to PrEP use. |
| Liboro, R., Despres, J., Ranuschio, B., Bell, S., Barnes, L. | 2021 | Canada | USA | Qualitative | Community based participatory research | Interviews | HIV/AIDs, Sexual or Gender Minorities | HIV-positive, middle-aged and older MSM on their individual attributes that helped forge their HIV/AIDS resilience | examine the perspectives and lived experiences of HIV-positive, middle-aged and older MSM on their individual attributes that helped forge their HIV/AIDS resilience |
| MacDonald, C., Martin, M. R., Steenbeek, A., Browne, A. | 2015 | Canada | Canada | Qualitative | Feminist theory, Participatory action research, Post-Colonial research | Interviews, Sharing or Talking Circles (i) | Cervical Cancer, HPV | Mi’kmaq women from Mi'kmaq communities | explore women’s experiences with Pap screening in two rural Mi’kmaq communities using a broader lens to conceptualize their health-care experiences. |
| MacDonald, T., Jackson, S., Charles, M-C., Periel, M., Jean-Baptiste, M-V., Salomon, A., Premilus, E. | 2018 | Haiti | Canada | Qualitative | Community based participatory research, Community based action research | Focus Group, Interviews, Personal Reflection | Maternal Health Services, Maternal Mortality | Women and Men from rural Haitian communities | explore determinants of maternal mortality in rural Haiti through Community-Based Action Research (CBAR), guided by the delays that lead to maternal death. |
| Markus, S. F. | 2012 | USA, AI | USA | Qualitative | Critical theory, Participatory action research | Photovoice/video, Storytelling/Yarning (i) | Adolescent Sexual Health, HIV/AIDs, Pregnancy, STIs/STDs | American Indian Youth | provide an example of a culturally responsive, community-based project for addressing social determinants of health in rural American Indian (AI) communities |
| Mawdsley, A., Ranville, F., Gurney, L., Borden, B., Pooyak, S., Shannon, K., Krusi, A. | 2021 | Canada | Canada | Qualitative | Community based participatory research | Art Creation, Narratives, Photovoice/video | HIV/AIDs | cis and transgender indigenous women living with HIV | understand how the criminalization of HIV nondisclosure shapes the lived experiences of HIV-related stigma, disclosure, and health service among cis and transgender Indigenous women living with HIV (IWLWH) |
| Mosley, E. A, Ayala, S., Jah, Z., Hailstorks, T., Dixon, D. D., Hernandez, N., Jackson, K., Hairston, I., Hall, K. S. | 2022 | USA | USA | Qualitative | Case study, Community based participatory research, Community-led research, Participatory action research | Focus Group, Interviews | Abortion | black and marginalized communities affected by SRH inequities | demonstrate the principles for community-led research in reproductive research along with the strengths and Challenges of reproductive justice research |
| Nguyen, T. C. K. Y., Lee, L., Frances, L., Michael, C., Gedaly, D. V., Nail, L. M., Wang, P., Ru, T. T. | 2012 | USA | USA | Quantitative | Descriptive, Ecological Study, CBPR | Survey/Questionnaire | Cervical Cancer, HPV | Vietnamese American immigrant Women | explore factors potentially influencing Pap testing practices among Vietnamese American immigrant women (VIW, foreign-born) and describe their awareness of cervical cancer screening resources in their community. |
| Oster, R. T., Bruno, G., Montour, M., Roasting, M., Lightning, R., Rain, P., Graham, B., Mayan, M. J., Toth, E. L., Bell, R. C. | 2016 | Canada | Canada | Qualitative | Community based participatory research, Ethnography | Interviews, Personal Reflection | Prenatal Health | Pre-natal healthcare Providers | explore the characteristics of effective care with First Nations women from the perspective of prenatal healthcare providers (HCPs). |
| Rand, J. R. | 2016 | Canada | Canada | Indigenous Research Methods | Post-Colonial research, 2 eyed seeing, Inuit Qaujimajatuqangit | Storytelling/Yarning (i) | HIV/AIDs, Sexual Health, STIs/STDs | Inuit Women | create a dialogue with Inuit women to address the lack of information available to inform programming to improve the sexual health of Inuit women, their families, and their communities in the Canadian Arctic |
| Rink, E., FourStar, K., Anastario, M. P. | 2017 | USA, AI | USA | Qualitative | Community based participatory research | Interviews | Contraception, Family Planning, HIV/AIDs, STIs/STDs | Heterosexual American Indian men living on a reservation | examine the relationship between American Indian men’s attitudes toward pregnancy prevention, STI/HIV prevention, and sexual risk behavior |
| Rink, E., Ricker, A., FourStar, K., Anastario, M. | 2016 | USA (Indian reservation) | USA | Mixed Methods | Community based participatory research, Quasi-Experimental | Interviews, Survey/Questionnaire | Reproductive Health, Sexual/Reproductive Education, Sexual Health | Heterosexual men from the Assiniboine or Sioux tribes | Investigate the effectiveness of a sexual and reproductive health peer led education intervention model for AI men, ages 18–24. |
| Santos Hovener, C., Marcus, U., Koscho,  l. C., Oudini, H., Wiebe, M., Ouedraogo, O. I., Thorlie, A., Bremer, V., Hamouda, O., Dierks, M. L., An Der, H. M,, Krause, G. | 2015 | Germany | Germany | Quantitative | Cross-sectional study | Survey/Questionnaire | HIV/AIDs, STIs/STDs (Viral Hepatitis) | Migrants from Sub-Saharan Africa | Discover information on knowledge, attitudes, behaviors and practices (KABP) regarding sexual health in African communities residing in Germany. |
| Shabangu, P. N., Brear, M. R. | 2017 | Eswatini | Swaziland | Qualitative | Participatory action research | Focus Group | Adolescent Sexual Health, HIV/AIDs, Sex Work | members of a rural Swazi community caring for children affected by AIDS | learn from members of a  rural Swazi community caring for children affected by AIDS,  about how gendered childcare norms and expectations  influence young women’s HIV risk and might be addressed by  innovative, structural approaches to HIV prevention. |
| Varcoe, C., Browne, A. J., Ford Gilboe, M., Dion Stout, M., McKenzie, H., Price, R., Bungay, V., Smye, V., Inyallie, J., Day, L., Khan, K., Heino, A., Merritt Gray, M. | 2017 | Canada | Canada | Qualitative | None Identified | Interviews | IPV/GBV | Indigenous women | describe the adaptation, pilot testing, and revision of a health promotion intervention for urban Indigenous women who have experienced IPV. |
| Wallace,,H, J., McDonald, S. Belton, S, Miranda, A, I., Costa, E. da Matos, L. C. Henderson, H. Taft, A. | 2018 | Timor-Leste | Australia | Indigenous Research Methods | Decolonized Methodology, two-eyed seeing | Body Mapping | Contraception, Family Planning, Reproductive Health | people of reproductive age in Timor-Leste | To demonstrate that body mapping is an effective  method to traverse language and culture to gain emic insights and indigenous worldviews, gain emic perspectives on maternal mortality and fertility, and determine what people know and believe about reproductive anatomy, physiology, and contraception. |
| Wallace, H. J., McDonald, S., Belton, S., Miranda, A. I., da  Costa, E., Matos, L. D. C., Henderson, H., Taft, A. | 2018 | Timor-Leste | Australia | Qualitative | Decolonized Methodology | Focus Group, Interviews | Antenatal, Birth | Timor-Leste men and women of reproductive age | identify what influences people’s decisions to seek antenatal care and care during labour and birth in Timor-Leste, a low-middle income newly independent nation in South East Asia with a high maternal death rate. The study aimed to provide emic/local insights to help midwives and maternal health providers tailor care and resources appropriately, thus improving maternal health. |
| Wilson, D., Mikahere Hall, A., Jackson, D., Cootes, K., Sherwood, J. | 2021 | New Zealand | New Zealand | Indigenous Research Methods | Kaupapa Maori | Interviews | IPV/GBV | Māori women | provide an overview of precolonial and contemporary  Māori values and the impact of colonization on Māori women’s intimate  partner relationships. To examine Māori women’s sociocultural constructions of “love” in relationships with violent partners and the roles traditional Māori values of aroha (compassion, empathy, and respect) and manaakitanga (hospitality, sharing, and caring for others) play in their relationships. |
| Wood, L., Hendricks, F. | 2017 | South Africa | South Africa | Qualitative | Participatory action research | Interviews, Narratives | Adolescent Sexual Health, Pregnancy | School-going youth in South Africa | involve school-going youth in the research and development of prevention interventions, tailored to meet their perceived needs |
| Yancey, E. M., Mayberry, R., Armstrong Mensah, E., Collins, D., Goodin, L., Cureton, S., Trammell, E. H., Yuan, K. | 2012 | USA | USA | Quantitative | Experimental (RCT) | Experiments/Clinical Trial, Survey/Questionnaire | HIV/AIDs | Heterosexually Active African American Men and  Women | design and test HIV-RAAP (HIV/AIDS Risk Reduction Among Heterosexually Active African American Men and Women: A Risk Reduction Prevention Intervention) a coeducational, culture- and gender-sensitive community-based participatory HIV risk reduction intervention. |
| Zehbe, I., Wood, B., Wakewich, P., Maar, M., Escott, N., Jumah, N., Little, J. | 2015 | Canada | Canada | Mixed Methods | Participatory action research, Experimental (RCT) | Focus Group, Interviews, Experiments/Clinical Trial | Cervical Cancer, HPV | First Nations women in Canada | explore educational strategies for engaging First Nations women in Canada to attend cervical cancer screening. |

Table S2: Study design, methodologies, and methods of all included articles

| **Study Design** | n | % |
| --- | --- | --- |
| *Qualitative* | 22 | 63% |
| *Indigenous Research Methods* | 6 | 17% |
| *Quantitative* | 3 | 9% |
| *Mixed Methods* | 4 | 11% |
| **Methodology** | n | % |
| *Community Based Participatory Research* | 13 | 37% |
| *Community-Led Research* | 2 | 6% |
| *Critical Theory* | 1 | 3% |
| *Decolonized Methodology* | 4 | 11% |
| *Ethnography* | 3 | 9% |
| *Feminist Theory* | 3 | 9% |
| *Grounded Theory* | 1 | 3% |
| *Participatory Action Research* | 13 | 37% |
| *Post-Colonial Research* | 4 | 11% |
| *None Identified* | 1 | 3% |
| *Focused Ethnography* | 1 | 3% |
| *Research Priorities of Affected Communities protocol* | 1 | 3% |
| *Cree research protocol and ethics* | 1 | 3% |
| *Mana Wāhine (Māori feminist)* | 1 | 3% |
| *Two-eyed seeing* | 2 | 6% |
| *Qaujimajatuqangit* | 1 | 3% |
| *Kaupapa Maori* | 1 | 3% |
| *Cross-Sectional study* | 2 | 6% |
| *Descriptive* | 1 | 3% |
| *Ecological Study* | 1 | 3% |
| *Experimental (RCT)* | 2 | 6% |
| *Quasi-Experimental* | 1 | 3% |
| *None identified* | 2 | 6% |
| **Qualitative/Indigenous Methods** | n | % |
| *Art Creation* | 1 | 3% |
| *Body Mapping* | 1 | 3% |
| *Culturally-Specific Methods (i)* | 1 | 3% |
| *Document Review* | 2 | 6% |
| *Field Notes* | 1 | 3% |
| *Focus Groups* | 11 | 31% |
| *Interviews* | 21 | 60% |
| *Narratives* | 3 | 9% |
| *Observation* | 2 | 6% |
| *Personal Reflection* | 2 | 6% |
| *Photovoice/Video* | 10 | 29% |
| *Storytelling/Yarning (i)* | 3 | 9% |
| *Sharing or Talking Circles (i)* | 2 | 6% |
| *Visiting (i)* | 1 | 3% |
| *Other* | 12 | 34% |
| **Quantitative Methods** | n | % |
| *Experiments/Clinical Trial* | 2 | 6% |
| *Survey/Questionnaire* | 6 | 17% |
| *Not Identified* | 1 | 3% |

Table S3: Methodologies of qualitative studies

| **Qualitative Research Methodology** | **%** | **#** |
| --- | --- | --- |
| **CBPR** | 50.0% | 11 |
| **PAR** | 40.9% | 9 |
| **Ethnography** | 13.6% | 3 |
| **Post-Colonial Research** | 13.6% | 3 |
| **Other** | 13.6% | 3 |
| **Feminist Theory** | 9.1% | 2 |
| **Case Study** | 4.5% | 1 |
| **Community-Led Research** | 4.5% | 1 |
| **Critical Theory** | 4.5% | 1 |
| **Decolonized Methodology** | 4.5% | 1 |
| **None Identified** | 4.5% | 1 |

Table S4: Methodologies of Indigenous research studies

| **Indigenous Research Methodology** | **%** | **#** |
| --- | --- | --- |
| **Other** | 83.3% | 5 |
| **Decolonized Methodology** | 50.0% | 3 |
| **CBPR** | 16.7% | 1 |
| **Community-Led Research** | 16.7% | 1 |
| **Feminist Theory** | 16.7% | 1 |
| **PAR** | 16.7% | 1 |
| **Post-Colonial Research** | 16.7% | 1 |

Table S5: Methodologies of mixed method studies

| **Mixed Methods Research Methodology** | **%** | **#** |
| --- | --- | --- |
| **PAR** | 75.0% | 3 |
| **CBPR** | 25.0% | 1 |
| **Grounded Theory** | 25.0% | 1 |
| **Cross-Sectional Study** | 25.0% | 1 |
| **Experimental (RCT)** | 25.0% | 1 |
| **Quasi-Experimental** | 25.0% | 1 |
| **None Identified** | 25.0% | 1 |

Table S6: Methodologies of quantitative studies

| **Quantitative Research Methodologies** | **%** | **#** |
| --- | --- | --- |
| **Cross-Sectional Study** | 33.3% | 1 |
| **Ecological Study** | 33.3% | 1 |
| **Experimental (RCT)** | 33.3% | 1 |
| **None Identified** | 33.3% | 1 |

Table S7: Definitions of elements of decolonizing principle "Challenging Western research foundations"

| **Elements of “Challenging Western Research Foundations”** | **Brief definition** |
| --- | --- |
| Inclusion of influences beyond dominant culture in development of methodology | Methodology identifies non-Western inspiration in developing research procedures |
| Acknowledge the use of Western paradigms | Expresses understanding that primary epistemology/ontology is based in Western or dominant culture norms |
| Question the use of Western paradigm as applicable | Questions if Western norms/epistemology are relevant to context |
| Joint creation of methodology with community | Co-creation of a unique or new methodology beyond the scope of dominant culture Western methodologies |
| Acknowledge epistemological or ontological assumptions | Recognizes assumptions or biases made about ways of knowing or being |
| Refers to transformative methodology | Identifies a methodology as one that will create a new concept or change understanding |

Table S8: Definitions of elements of decolonizing principle "Critiquing power structures"

| **Elements of “Critiquing Power Structures”** | **Brief definition** |
| --- | --- |
| Directly acknowledges colonialism as an element of its methodology | Methodology explicitly mentions the impact of colonialism in developing methodology or on outcome of research |
| Directly acknowledges sexism/patriarchy as an element of its methodology | Methodology explicitly mentions the impact of sex/gender in developing methodology or on outcome of research |
| Directly acknowledges racism/race as an element of its methodology | Methodology explicitly mentions the impact of race in developing methodology or on outcome of research |
| Directly acknowledges SES/economic disadvantage as an element of its methodology | Methodology explicitly mentions the impact of SES in developing methodology or on outcome of research |
| Directly acknowledges heteronormativity as an element of its methodology | Methodology explicitly mentions the impact of heteronormativity in developing methodology or on outcome of research |
| Directly acknowledges ableism as an element of its methodology | Methodology explicitly mentions the impact of ableism in developing methodology or on outcome of research |
| Directly acknowledges other situationally specific power imbalance/tools of oppression/hierarchical systems | Methodology explicitly mentions the impact of power imbalances in developing methodology or on outcome of research |
| Includes bias or cultural training as an element of methodology | Bias training included as research procedure |
| Acknowledge cultural competency/positionality/insider-outsider status | Methodology references researcher positionality |
| Acknowledges the power imbalance between researcher and researched | Methodology references specific power differential between researcher and community |
| Research as reciprocal | Identifies equal distribution of power/knowledge/results between the community and the researcher |

Table S9: Definitions of elements of decolonizing principle "Centering the community"

| **Elements of “Centering the Community”** | **Brief definition** |
| --- | --- |
| Recognizes the self-identification of a “community” | Participants of the study self-identify as belonging to the relevant “community” |
| Recruitment strategies to center relevant community members | Specific recruitment measures identified designed for relevant individuals |
| Community voices are engaged as a part of the methodology | The direct acknowledgement of an involvement of the community within the methodology section e.g. community advisory board |
| Strengths based approach to the community | Methodology uses community skills and values as part of design |
| Promotes co-learning with community in some way | General collaboration in the construction of knowledge identified |
| Equity based approach to learnings/knowledge | Methodology balances ownership of research output with interests of the community |
| Community-defined problems | Research question or issue are defined or discussed with community before research begins |
| iterative, co-learning process | Multiple points of communication and member-checking referenced |
| open partnership with community | Community is engaged as an equal partner in methodology |
| Commitment to articulating diverse voices | Diversity specific accommodations are made to ensure equitable research |
| Individuals centred/ allowed to speak for themselves | Research identifies unique strategies where community members speak independently rather than as response to specific prompt |

Table S10: Web of Science Boolean Operators

(ALL=(decoloni* research OR community-based participatory research or citizen science OR community based research OR indigenous research OR participatory action research OR community-based research OR culturally grounded OR community-led research OR culturally sensitive research OR grounded theory OR reproductive justice OR photovoice OR grassroots research OR participatory visual methods OR community mapping)) AND ALL=(sexual health OR family planning OR Pregnancy OR Sexually transmitted OR Gender based violence OR spousal abuse OR HIV OR abortion OR sexual and gender minorities OR menstruation OR Contracept* OR Reproductive health OR HPV OR female genital mutilation OR clitoral cutting OR herpes or chlamydia OR syphilis OR gonorrhea OR fertility OR sexual health education OR condoms OR birth control OR sexual wellness OR child marriage OR sexual behaviour OR safe sex OR masturbation OR sex work OR infertility )
